# Metformin Inhibits the Urea Cycle and Reduces Putrescine Generation in Colorectal Cancer Cell Lines

**DOI:** 10.3390/molecules26071990

**Published:** 2021-04-01

**Authors:** Tao Zhang, Ling Hu, Jia-Feng Tang, Hang Xu, Kuan Tian, Meng-Na Wu, Shi-Ying Huang, Yu-Mei Du, Peng Zhou, Rui-Jin Lu, Shuang He, Jia-Mei Xu, Jian-Jun Si, Jing Li, Di-Long Chen, Jian-Hua Ran

**Affiliations:** 1 Department of Anatomy, College of Basic Medicine, Chongqing Medical University, Chongqing 400016, China; tao5227@sina.cn (T.Z.); huling199418@163.com (L.H.); xuhang1996719@163.com (H.X.); tiankuan927@163.com (K.T.); wumengna@yeah.net (M.-N.W.); xujiamei1232021@163.com (J.-M.X.); s771489334@163.com (J.-J.S.); 2 Lab of Stem Cell and Tissue Engineering, Department of Histology and Embryology, Chongqing Medical University, Chongqing 400016, China; tjf@stu.cqmu.edu.cn (J.-F.T.); HuangSY0928@163.com (S.-Y.H.); duyumei111@163.com (Y.-M.D.); zp979391906@163.com (P.Z.); lrj18375757010@163.com (R.-J.L.); zth@stu.cqmu.edu.cn (S.H.); 100392@cqmu.edu.cn (J.L.); xinmengyuandlc@163.com (D.-L.C.); 3 Chongqing Three Gorges Medical College, Chongqing Engineering Research Center of Antitumor Natural Drugs, Chongqing 404120, China

**Keywords:** metformin, colorectal cancer (CRC), p53, proliferation, urea cycle, human colorectal cancer HCT116 cell line, CPS1, ARG1, OTC, ODC, putrescine

## Abstract

The urea cycle (UC) removes the excess nitrogen and ammonia generated by nitrogen-containing compound composites or protein breakdown in the human body. Research has shown that changes in UC enzymes are not only related to tumorigenesis and tumor development but also associated with poor survival in hepatocellular, breast, and colorectal cancers (CRC), etc. Cytoplasmic ornithine, the intermediate product of the urea cycle, is a specific substrate for ornithine decarboxylase (ODC, also known as ODC1) for the production of putrescine and is required for tumor growth. Polyamines (spermidine, spermine, and their precursor putrescine) play central roles in more than half of the steps of colorectal tumorigenesis. Given the close connection between polyamines and cancer, the regulation of polyamine metabolic pathways has attracted attention regarding the mechanisms of action of chemical drugs used to prevent CRC, as the drug most widely used for treating type 2 diabetes (T2D), metformin (Met) exhibits antitumor activity against a variety of cancer cells, with a vaguely defined mechanism. In addition, the influence of metformin on the UC and putrescine generation in colorectal cancer has remained unclear. In our study, we investigated the effect of metformin on the UC and putrescine generation of CRC in vivo and in vitro and elucidated the underlying mechanisms. In nude mice bearing HCT116 tumor xenografts, the administration of metformin inhibited tumor growth without affecting body weight. In addition, metformin treatment increased the expression of monophosphate (AMP)-activated protein kinase (AMPK) and p53 in both HCT116 xenografts and colorectal cancer cell lines and decreased the expression of the urea cycle enzymes, including carbamoyl phosphate synthase 1 (CPS1), arginase 1 (ARG1), ornithine trans-carbamylase (OTC), and ODC. The putrescine levels in both HCT116 xenografts and HCT116 cells decreased after metformin treatment. These results demonstrate that metformin inhibited CRC cell proliferation via activating AMPK/p53 and that there was an association between metformin, urea cycle inhibition and a reduction in putrescine generation.

## 1. Introduction

Colorectal cancer (CRC) is the third most commonly diagnosed cancer in males and the second most common malignancy in females. New cases exceeded 1.8 million and approximately 861,000 deaths were reported in 2018 [1]. The prognosis of CRC varies according to the cancer stage, with a five-year survival rate of about 90% for stage I disease and a dismal ~10% rate for stage IV patients. Although 60% of patients present with a resectable disease at the time of diagnosis, nearly half of these patients who undergo curative surgery, and another 20–25% who receive post-surgical adjuvant chemotherapy, experience cancer relapse, metastatic disease, and eventual death [2,3,4]. Similarly, the use of medicine for the chemoprevention of colorectal cancer is limited due to the possible adverse side effects associated with their long-term use [5,6], which highlights the urgency of improving the current state of treatment choices for this fatal malignancy. 

Cancer cells constantly alter different metabolic pathways to meet their ever-changing metabolic needs determined by changes in the cell and environment [7]. Reducing catabolism and nitrogen processing is a good strategy in this regard. The urea cycle (UC), discovered by Krebs and Henseleit [8,9], removes the excess nitrogen and ammonia generated by nitrogen-containing compound composites or protein breakdown in the human body [10,11].

In the liver, the UC is coordinated by five catalytic enzymes: ornithine trans-carbamylase (OTC) and carbamoyl phosphate synthase 1 (CPS1) are mitochondrial enzymes, and arginino-succinate lyase (ASL), arginino-succinate synthase (ASS1), and arginase1 (ARG1) are cytoplasmic enzymes. Increasingly, research is showing that changes in urea cycle enzymes are not only related to tumorigenesis and tumor development but also associated with poor survival in hepatocellular, breast, and colorectal cancers, etc. [12,13,14,15,16]. 

Cytoplasmic ornithine, the intermediate product of the urea cycle, is a specific substrate of ornithine decarboxylase (ODC) for the production of putrescine and is required for tumor growth [17]. Polyamines (spermidine, spermine, and their precursor putrescine) are polycationic compounds that play central roles in more than half of the steps of colorectal tumorigenesis. In addition, polyamines are signs of tumor proliferation in the colon. Among the many biochemical alterations that have been found in cancer cells, a change in cellular polyamine content is one of the most consistent. 

The concentrations of polyamines are increased during the formation of tumors, and the activity of ODC is increased with neoplastic transformation [18]. As with other tumors, CRCs show a higher polyamine density than equivalent normal tissues and neighboring mucosa [19,20]. In light of the close connection between polyamines and cancer, the regulation of polyamine metabolic pathways (such as the de novo synthesis and catabolism of polyamines) has attracted much attention regarding the potential use of chemical drugs to prevent human colorectal cancer [21].

The drug most widely used to treat type 2 diabetes (T2D) [22], metformin exhibits antitumor activity against a variety of cancer cells, including those of colorectal cancer [23]. Previous research results indicated that the influence of metformin on cancer cells is related to the activation of monophosphate (AMP)-activated protein kinase (AMPK) [24]. The activation of AMPK is related to a variety of cellular incidents, including the inhibition of cell growth and blood vessel formation, and the induction of cell cycle arrest, fatty acid synthesis, autophagy, and apoptosis [25]. Although the inhibitory influence of metformin on tumors has been confirmed, the molecular details of this process have not been discovered [22]. Recent research showed that metformin-mediated AMPK activation can activate p53 [26].

p53 is a linchpin molecule that participates in multiple cellular procedures, such as cell cycle arrest, aging, and apoptosis, to achieve the purpose of preventing tumor formation [27]. Recent studies have indicated additional p53 functions largely dedicated to tumor repression. p53-mediated metabolic rearrangement has been demonstrated to play a central role in the tumor suppression mediated by p53 [28,29]. Recent research revealed that p53 exhibits inhibitory effects on tumor proliferation by regulating the urea cycle [30]. Thus, we assume that metformin exerts anti-colorectal carcinoma functions by downregulating the urea cycle and inhibiting polyamine biosynthesis via the activation of p53.

In the present study, to determine the function of metformin in the growth inhibition of colorectal cancer and the underlying mechanism, we investigated the effect of metformin on the proliferation of colorectal cancer cells and detected changes in the urea cycle and polyamine biosynthesis pathway in vivo and in vitro. 

After treatment with metformin, the cell cycle of CRC cells was significantly arrested at the G1 phase; the expression of the AMPK, p53, and p21 proteins was upregulated; the expression of the CPS1, ARG1, OTC, and ODC proteins was downregulated; and the putrescine levels in HCT116 xenografts and HCT116 cells were decreased. The inhibition of the urea cycle leads to the accumulation of ammonia [30]. NH_4_Cl treatment was shown to have the same inhibitory influence as metformin on the cellular proliferation of colorectal cancer. 

Treatment with the p53 activator nutlin-3 showed inhibitory effects similar to those of metformin treatment on the cellular proliferation of colorectal cancer. Metformin rescued the p53-inhibitory effect mediated by pifithrin-α (5 μM). Our results demonstrated that metformin inhibited colorectal cancer cell proliferation via activating AMPK/p53. The mechanism may involve downregulating the urea cycle enzymes and reducing polyamine biosynthesis. Metformin can be used as a therapeutic agent for human colorectal cancer.

## 2. Results

### 2.1. Metformin Reduced the Expression of ODC and Inhibited the Growth of Colorectal Cancer Cells In Vivo 

**Figure 1:**
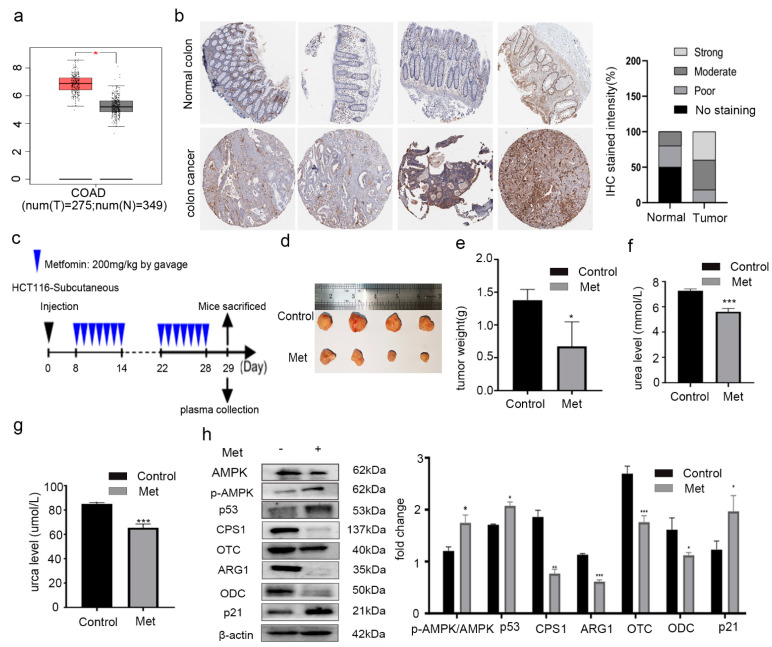
Metformin (Met) reduced the expression of ornithine decarboxylase (ODC) and inhibited the growth of colorectal cancer cells in vivo. (**a**) The expression of ODC1 in colon adenocarcinoma (COAD). The data were generated from Gene Expression Profiling Interactive Analysis (GEPIA). (**b**) The intensity of staining in normal colon and in colon cancer tissues according to the human protein atlas (HPA) database and statistical analysis. (**c**) Schematic of the treatment protocol for metformin in the HCT116 xenograft model. (**d**) Typical images of the tumors from the control and metformin-treatment groups. (**e**) The tumor weight decreased in metformin-treatment mice. The levels of (**f**) urea and (**g**) urca decreased in the metformin group. (**h**) Decreased expression levels of proteins in the urea cycle and putrescine biosynthesis pathway in tumor tissues were detected using Western blotting. Values are the means and standard errors of three separate experiments (* *p* < 0.05, ** *p*<0.005, and *** *p* < 0.001, two-tailed Student’s *t*-tests).

First, we measured the expression of ODC1 (Figure 1a) in colorectal cancer by Gene Expression Profiling Interactive Analysis (GEPIA) (http://gepia.cancer-pku.cn/; accessed on 16 February 2021). The expression of ODC1 in colorectal cancer was significantly higher than that in the normal colon tissues. In addition, we further determined, using the human protein atlas (HPA) database (https://www.proteinatlas.org/; accessed on 16 February 2021), that the staining intensity for ODC1 in normal colon tissues was either poor or nonexistent, whereas the intensity in colon cancer tissues was mostly moderate or strong (Figure 1b), which suggested that a change in the ODC1 protein could be a target for the chemoprevention of colorectal cancer.

Next, we investigated whether metformin could inhibit the growth of colorectal cancer cells in vivo. As shown in Figure 1c–e, metformin exhibited inhibitory effects on tumor growth in vivo, with smaller tumor volumes and decreased tumor weights observed in metformin-treated mice compared to the control group. The biochemical analysis showed that the urea (Figure 1f) and urca levels significantly decreased (Figure 1g) in the metformin-treatment group compared with the control.

The results suggest that the urea cycle in the metformin-treatment group may have been inhibited. To verify whether the inhibitory effect of metformin on xenograft tumor growth was mediated by inhibiting the urea cycle and decreasing ODC1 levels, the levels of AMPK, p-AMPK, p53, ARG1, CPS1, OTC, p21, and ODC in HCT116 xenografts were assayed using Western blotting. Increased protein levels of AMPK, p-AMPK/AMPK, p53, and p21 and decreased levels of CPS1, ARG1, OTC, and ODC (Figure 1h) were detected in the metformin-treatment group compared with the control.

### 2.2. Metformin Reduced Putrescine Levels and Urea Cycle Intermediates in HCT116 Xenografts

**Figure 2:**
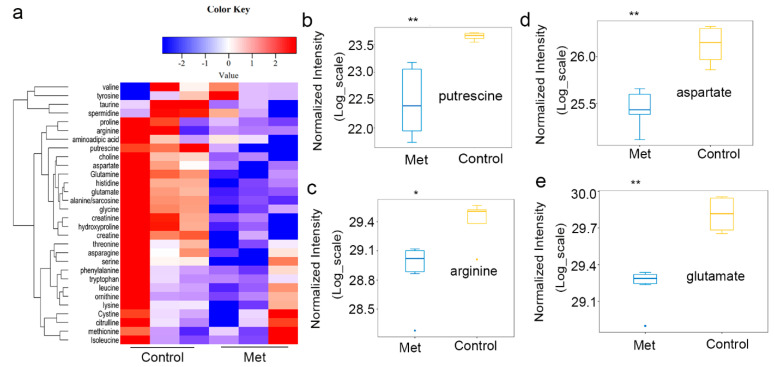
Metformin reduced the putrescine and urea cycle intermediates in HCT116 xenografts. In light of the in vivo results showing that metformin can change the expression of urea cycle proteins and ODC1 in HCT116 xenografts, the amino acid metabolism of those xenografts was examined. (**a**) Cluster diagram of the metabolic changes; the levels of (**b**) putrescine, (**c**) arginine, (**d**) aspartate, and (**e**) glutamate were quantified by liquid chromatography coupled to tandem mass spectrometry (LC–MS/MS). Values are the means and standard errors of four separate experiments (* *p* < 0.05, ** *p* < 0.005, two-tailed Student’s *t*-tests).

In light of the in vivo results showing that metformin altered the expression of urea cycle enzymes and ODC1 in HCT116 xenografts, the amino acid metabolism in those xenografts was examined. The levels of putrescine and urea cycle intermediates decreased (Figure 2a), such as those for putrescine (Figure 2b), arginine (Figure 2c), aspartate (Figure 2d), and glutamate (Figure 2e), in HCT116 xenografts treated with metformin. These results demonstrated that metformin inhibited the production of intermediates in the urea cycle and putrescine biosynthesis in vivo.

### 2.3. Metformin Inhibited Cell Viability by Inducing G1 Phase Cell Cycle Arrest in Colorectal Cancer Cells

**Figure 3:**
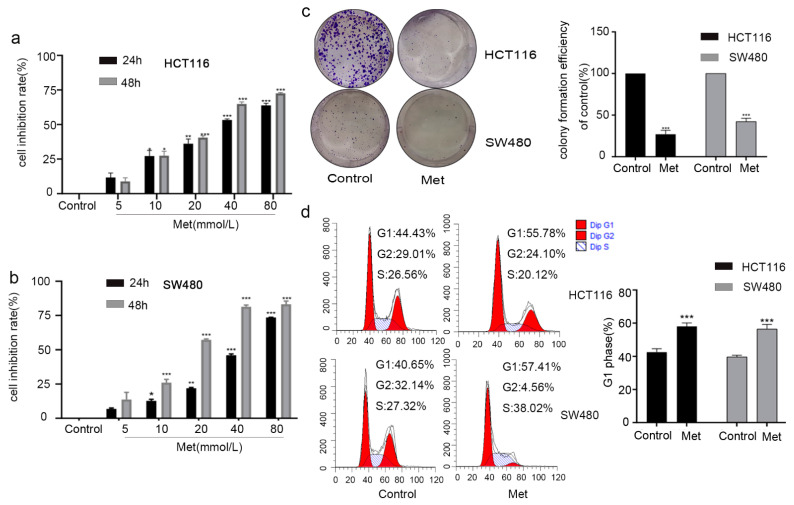
Metformin inhibited cell viability and induced G1 phase arrest in colorectal cancer cells in vitro. The cytotoxic effect of metformin on (**a**) HCT116 cells and (**b**) SW480 cells as detected by Cell Counting Kit-8 (CCK-8) assay. (**c**) The cell cycle of HCT116 and SW480 cells detected by propidium iodide staining and flow cytometry. (**d**) Representative images of the colony-formation assay. Values are the means and standard errors of three separate experiments (* *p* < 0.05, ** *p* < 0.005, and *** *p* < 0.001, two-tailed Student’s *t*-test).

Next, we explored the anti-proliferative effect of metformin on HCT116 (Figure 3a) and SW480 (Figure 3b) cells, which were treated with increasing concentrations (0, 5, 10, 20, 40, and 80 mM) of metformin for 24 and 48 h in a Cell Counting Kit-8 (CCK-8) assay. The viability of the HCT116 (Figure 3a) and SW480 (Figure 3b) cells was dramatically decreased by treatment with metformin for 24 and 48 h. Flow cytometry indicated that the G1 phase ratio of HCT116 and SW480 cells was enhanced after exposure to metformin for 24 h (Figure 3c). 

A long-term inhibitory effect of metformin on the colony formation of HCT116 and SW480 cells was confirmed by treating them with 20 mM metformin for 16 days (Figure 3d). In summary, these data indicate that the inhibitory effect of metformin on the growth of HCT116 and SW480 cells is related to cell cycle arrest in the G1 phase.

### 2.4. Metformin Inhibited the Proliferation of HCT116 Cells via Decreasing the Level of Putrescine and Increasing the Levels of Branched-Chain Amino Acids (BCAAs: Val, Ile, and Leu) 

**Figure 4:**
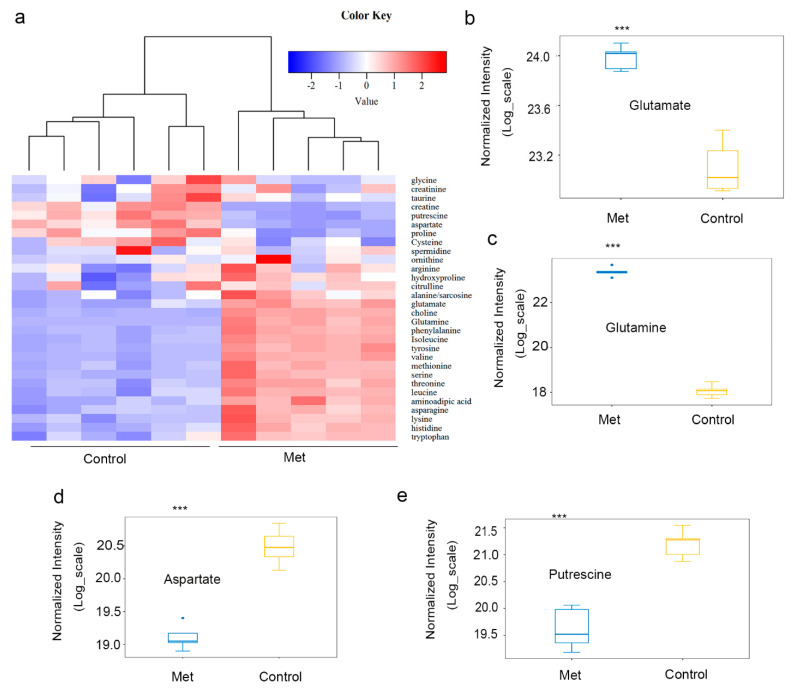
Metformin inhibited the proliferation of HCT116 cells via decreasing the level of putrescine and increasing the levels of Branched-Chain Amino Acids (BCAAs). The in vivo results indicate that metformin can change the levels of putrescine and urea cycle intermediates in HCT116 xenografts. The abundance of amino acids and putrescine in HCT116 cells treated with metformin (0 or 20 mM) for 24 h was quantified by LC–MS/MS. (**a**) Cluster diagram of the metabolic changes. The levels of (**b**) glutamate, (**c**) glutamine, (**d**) aspartate, and (**e**) putrescine were quantified by LC–MS/MS. Values are the means and standard errors of five separate experiments (*** *p* < 0.001, two-tailed Student’s *t*-tests).

Since metformin treatment could cause the downregulation of ODC expression in both HCT116 xenografts and colorectal cancer cells, amino acid metabolism was analyzed to assess a potential mechanism by which metformin treatment resulted in such downregulation in colorectal cancer cells. After the treatment of HCT116 cells with metformin, the level of putrescine decreased, while the levels of citrulline and branched-chain amino acids (BCAAs) increased (Figure 4a). Consistent with previous results, putrescine responded sensitively to alterations in polyamine synthesis, while spermidine and spermine were almost unaffected [31,32]. 

The increasing glutamate (Figure 4b) and glutamine (Figure 4c) levels, as well as the decreasing levels of aspartate (Figure 4d) and putrescine (Figure 4e), in HCT116 cells treated with metformin were analyzed. In rapidly proliferating cells, glutamine is used as a carbon source to supply the TCA cycle to sustain cellular bioenergetics and anabolic reactions. Previous research results indicated that, in cancer patients, certain tumors consume large amounts of glutamine, thereby reducing glutamine levels [33,34]. Due to the metabolic changes in HCT116 cells after metformin treatment, metformin inhibits cell proliferation via decreasing the level of putrescine and increasing the levels of BCAAs.

### 2.5. Metformin Inhibited Proliferation of Colorectal Cancer Cells by Changing Expression of AMPK and Urea Cycle Enzymes

**Figure 5:**
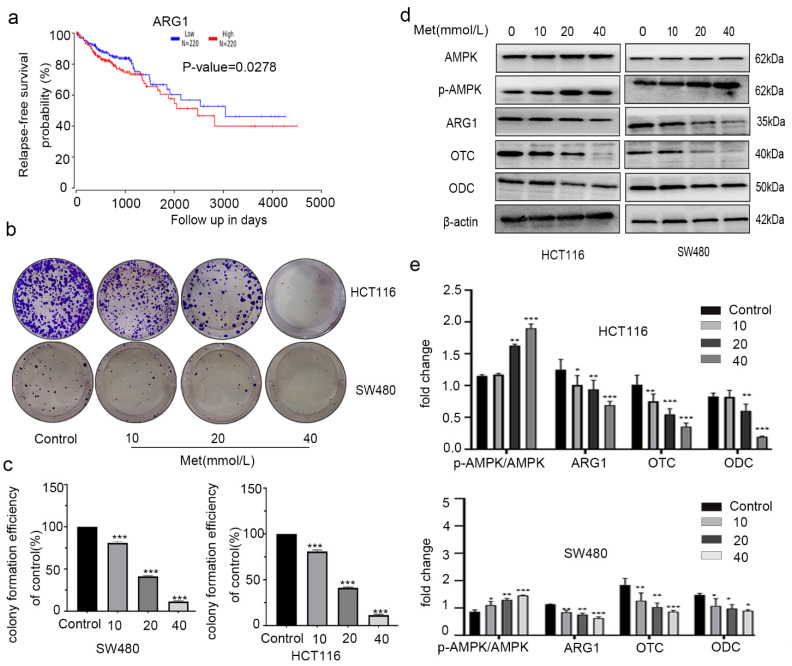
Metformin inhibited the proliferation of colorectal cancer cells by changing expression of monophosphate-activated protein kinase (AMPK) and the urea cycle. Kaplan–Meier survival curves for patients with colorectal cancer according to arginase 1 (ARG1) (**a**) expression. The data were generated from the cancer genome atlas (TCGA) survival data. (**b**,**c**) Colony-formation assay. (**d**,**e**) The levels of AMPK, p-AMPK, and the urea cycle enzymes and ODC proteins in treated and untreated cells measured by Western blotting. Values are the means and standard errors of three separate experiments (* *p* < 0.05, ** *p* < 0.005, and *** *p* < 0.001, two-tailed Student’s *t*-tests).

The poor survival of colon cancer patients is related to a higher expression of ARG1 (Figure 5a) [35]. To explore the mechanism of metformin’s inhibition of the proliferation of colorectal cancer cells, HCT116 and SW480 cells were treated with increasing concentrations of metformin (0, 10, 20, and 40 mM) for 24 h. The colony-formation results show that metformin decreased the proliferation of HCT116 and SW480 cells (Figure 5b–c) in a concentration-dependent manner. 

The levels of AMPK, p-AMPK, and proteins in the urea cycle and putrescine generation pathways were assessed by Western blotting to explore the mechanism of metformin’s activity. The expression of p-AMPK/AMPK was significantly increased, and the protein levels of ARG1, OTC, and ODC were decreased in both HCT116 and SW480 cells (Figure 5d–e), in a dose-dependent manner. 

### 2.6. Metformin Inhibited Colorectal Cancer HCT116 Cell Proliferation by Activating p53

**Figure 6:**
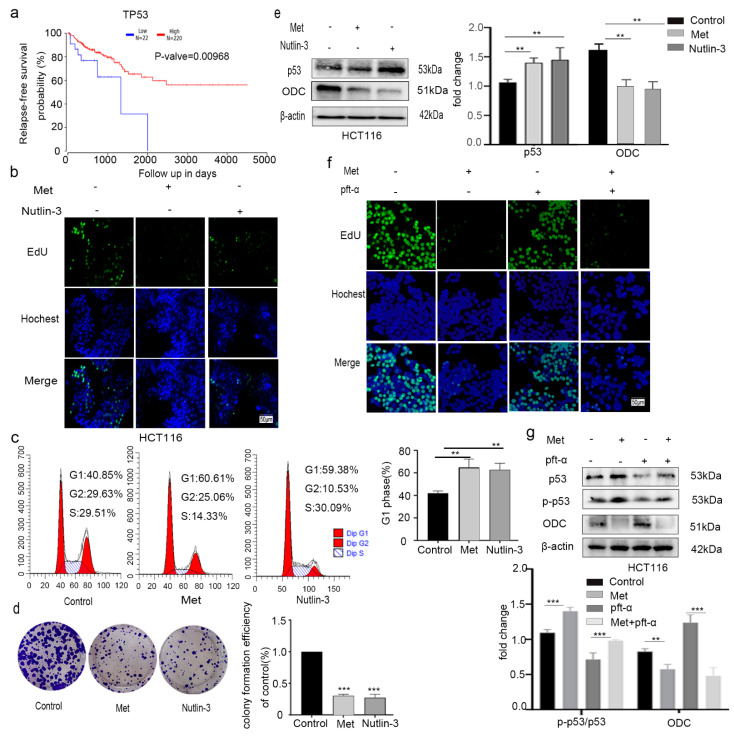
Metformin inhibited HCT116 cell proliferation by activating p53. Kaplan–Meier survival curves for patients with colorectal cancer according to TP53 (**a**) expression. The data were generated from the cancer genome atlas (TCGA) survival data. HCT116 cells were treated with Met (20 mM) or Nutlin-3 (5 µM) for 24 h. (**b**) 5-Ethynyl-2′-deoxyuridine (EdU) (x20) results; scale bar, 50 μm. (**c**) Cellular propidium iodide (PI) fluorescence, (**d**) colony-formation assay, and (**e**) the levels of p53 and ODC proteins in treated and untreated cells as measured by Western blotting. HCT116 cells were treated with 5 μM pifithrin-α for 24 h or not before exposure to 20 mM metformin. (**f**) EdU (x20); scale bar, 50 μm. (**g**) The levels of p53, p-p53, and ODC proteins in treated and untreated cells measured by Western blotting. The ratio of proliferating cells (EdU^+^) was calculated using ImageJ software (National Institutes of Health, Bethesda, MD, USA). Values are the means and standard errors of three separate experiments (** *p* < 0.005, and *** *p* < 0.001, two-way ANOVA followed by Tukey’s multiple comparisons).

In the current study, the expression of p-AMPK/AMPK was significantly increased in CRC cells after metformin treatment, indicating that metformin could induce AMPK activation. Saraei et al. showed that metformin-mediated AMPK activation can activate p53 [26]. The poor survival of colon cancer patients is related to the lower expression of p53 (Figure 6a), which shows that p53 activation helped to inhibit colorectal cancer. To validate whether metformin inhibited proliferation through p53 activation, Nutlin-3, a p53 activator, was administrated to HCT116 cells for 24 h and compared with metformin in its effects. 

The proliferation-inhibiting effect on colorectal cancer HCT116 cells induced by 20 mM metformin treatment was consistent with that of 5 µM Nutlin-3 as measured by incorporation of 5-ethynyl-2′-deoxyuridine (EdU) experiments (Figure 6b), a cycle arrest (Figure 6c) assay, and a colony-formation assay (Figure 6d). The higher expression of p53 and lower expression level of ODC were determined by Western blotting (Figure 6e). 

To further validate these results, pifithrin-α (pft-α, 0 or 5 μM), an inhibitor of p53′s transcriptional activity, was administered to HCT116 cells for 24 h before metformin (0 or 20 mM) treatment. Pft-α did not exhibit an inhibitory effect on the cell proliferation compared with the control according to EdU measurements; however, the cell proliferation was inhibited by treatment with metformin or pft-α combined with metformin (Figure 6f). The levels of the p53, p-p53 and ODC proteins were determined through Western blotting (Figure 6g).

Since the results for treatment with a p53-specific agonist were consistent with metformin-induced proliferation inhibition and cycle arrest, metformin rescued the p53 inhibition caused by pft-α, which indicates that metformin can inhibit colon cancer HCT116 cell proliferation by activating p53.

### 2.7. NH_4_Cl Suppressed the Proliferation of Colorectal Cancer Cells and Expression of ODC In Vitro 

**Figure 7:**
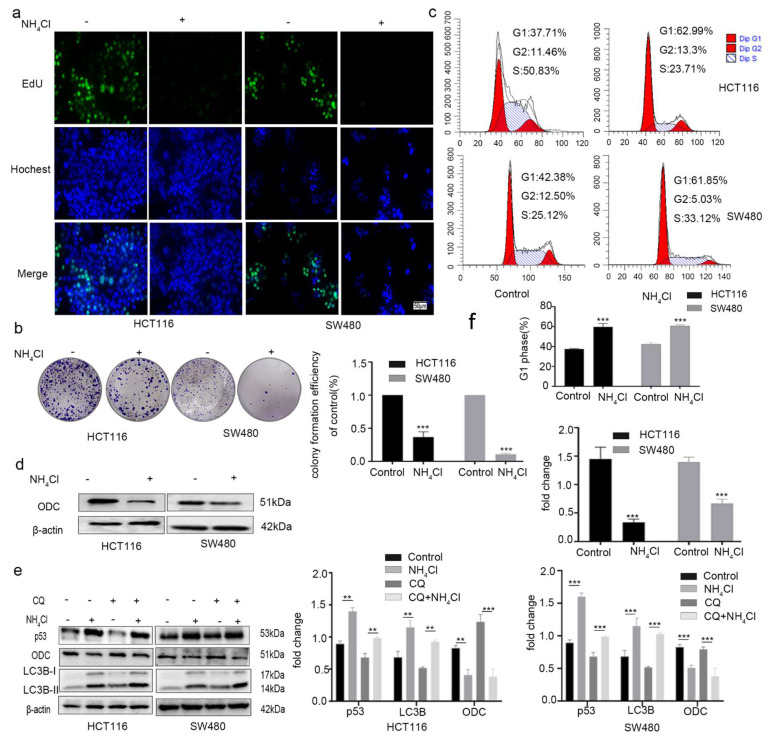
NH_4_Cl suppressed the proliferation of colorectal cancer cells and expression of ODC protein in vitro. HCT116 and SW480 cells were incubated with NH_4_Cl (5 mM) for 24 h. (**a**) EdU (x20) results. Scale bar, 50 μm. (**b**) Colony-formation assay, (**c**) cellular propidium iodide (PI) fluorescence, and (**d**) the levels of ODC protein in each group of cells as detected by Western blot methods. To exclude the effect of autophagy, 10 μM chloroquine (CQ), an inhibitor of autophagosome–lysosomal fusion, was used to pre-treat HCT116 cells (**e**) and SW480 cells (**f**) for 24 h before NH_4_Cl was added. The levels of p53, ODC, and LC3B proteins were measured by Western blotting. Values are the means and standard errors of three separate experiments (** *p* < 0.01, and ***p < 0.001; two-tailed Student’s t-test (**b**−**d**), or two-way ANOVA followed by Tukey’s multiple comparisons (**e**–**f**)).

Inhibiting the urea cycle increases ammonia levels [30]; ammonia replenishment reduces the growth of HCT116 and SW480 cells. To examine the effect of ammonia accumulation on the proliferation of colorectal cancer cells, NH_4_Cl, at 5 mM [30], was used to treat HCT116 and SW480 cells for 24 h. The proliferation of the HCT116 and SW480 cells was remarkably slowed when they were incubated with NH_4_Cl for 24 h (Figure 7a). 

NH_4_Cl had a clear inhibitory effect on the colony formation of HCT116 and SW480 cells relative to the untreated controls (Figure 7b). Flow cytometry (Figure 7c) revealed that the G1 phase ratio of HCT116 and SW480 cells increased after treatment with NH_4_Cl for 24 h. To provide further insight, Western blotting was used to determine the levels of the ODC protein in HCT116 and SW480 cells treated with 5 mM NH_4_Cl for 24 h (Figure 7d). 

Cheong et al. revealed that ammonia induced autophagy [36] and that metformin could also induce autophagy [37]. To remove the influence of autophagy, 10 μM chloroquine (CQ), an inhibitor of autophagosome–lysosomal fusion, was used to pre-treat HCT116 cells and SW480 cells for 24 h before NH_4_Cl was added. The expression of the p53, ODC, and microtubule-associated protein light chain 3β (LC3B) proteins changed consistently with the effects of metformin treatment, as measured by Western blotting (Figure 7e,f). Taken together, these data show that NH_4_Cl could suppress the proliferation of colorectal cancer cells and downregulate the expression of ODC in vitro.

## 3. Discussion

Even though clinicians have successfully decreased the mortality related to colorectal cancer through removing precursor lesions [38], CRC remains the third most common carcinoma in Western countries, and its occurrence is quickly rising, particularly in Asian countries [1,39]. Therefore, the exploration of novel therapeutic drugs with less toxicity is urgent. 

ODC is critical for the generation of putrescine from ornithine, and changes in ornithine metabolism in the UC affect the effectiveness of polyamine biosynthesis, thereby influencing proliferative programs [40]. The need for ODC activity (and the production of putrescine) in cell proliferation has been demonstrated in tissue research [41]. ODC induction and elevated polyamine levels have been reported in breast cancer [42] and prostate cancer [43]. 

The inhibition of ODC leads to decreased contents of putrescine, which is the precursor to spermidine and spermine—products of polyamine biosynthesis that are known for their functions in cell proliferation [44,45]. As the comprehension of the pathways affected by polyamines and the underlying molecular mechanisms by which polyamines are involved has advanced, the potential for developing the polyamine metabolic pathway as a scheme for cancer prevention and therapy has improved. Difluoro-methyl-ornithine(DFMO) is an inhibitor of the ODC effect that is believed to inhibit the spread of CRC cells and fast-growing colonic adenoma through polyamine consumption [46,47]. In phase IIb/III clinical trials, DFMO combined with sulindac, a nonselective inhibitor of cyclooxygenase-1(COX-1) and COX-2, effectively reduced the numbers of ectopic colorectal adenomas in individuals with histories of adenoma [48]. Although these results are inspiring, more clinical trials are required to investigate the efficacy and toxicity of DFMO in treating disease. With the characteristics of a good safety profile and low cost, metformin is a first-line treatment for T2D [22]. However, apart from being effective as a glucose-lowering agent, metformin has recently obtained more attention for its latent antitumorigenic effects [49]. In our study, after treatment with metformin, tumor growth was inhibited, and the results of the blood biochemical analysis of HCT116 xenograft mice in the Met group showed that urea and uric acid decreased, suggesting that the UC was inhibited. 

In addition, the expression of the ODC protein in both HCT116 cells and HCT116 xenografts was decreased (Figure 1h; Figure 5d), which resulted in a decrease in putrescine levels (Figure 2a,b; Figure 4a,e). These findings suggest that metformin may exert anti-colorectal carcinoma activity via the inhibition of the ODC.

Previous studies demonstrated that metformin may inhibit mammalian target of rapamycin (mTOR) through activating the liver kinase B1 (LKB1)/AMPK pathway; it may suppress the synthesis of proteins, downregulate the level of circulating insulin, restrain the reply of unfolded proteins, eliminate cancer stem cells, activate the immune system, or cause cell cycle arrest and/or apoptosis [49,50]. There is strong evidence that the activation of AMPK in vitro and in vivo is associated with the upregulation of T172 phosphorylation. In our study, increased expression of p-AMPK(T172) was detected both in vivo and in vitro after metformin treatment, which suggests that metformin could induce AMPK activation. AMPK regulates p53 expression and phosphorylation [51,52,53]. AMPK activation in HepG2 cells, which were derived from human hepatocellular carcinoma carrying wild-type p53, led to G1 cell cycle arrest through stabilizing p53 [54]. The phosphorylation of p53 at Ser-15 is important for AMPK-mediated cell cycle arrest in response to low glucose. The stress-induced phosphorylation of p53 at Ser-15 could disrupt the p53–MDM2 interaction, enhancing p53′s activity and stability [55,56,57]. Metformin may inhibit mTOR through activating the LKB1/AMPK pathway, which could activate p53 and induce cell cycle arrest [58]. According to the results in the present research, metformin suppressed cell proliferation and caused cell cycle arrest at G1, which shows that the AMPK activation was mediated by metformin induced p53 activity. Nutlin-3, an inhibitor of the MDM2–p53 interaction, inhibits MDM2′s binding to p53 and stabilizes p53 [59]. The effects of Nutlin-3 treatment were consistent with metformin-induced proliferation inhibition and cycle arrest. Metformin rescued the inhibition of p53 by pft-α, indicating that metformin induced p53 activation. 

p53, a long-established tumor suppressor, plays a central role in the cellular reactions in a variety of stress semaphores, including DNA damage, hypoxia, and the activation of oncogenic pathways [60]. Its activation can cause a series of reactions ranging from cell cycle arrest or DNA repair to apoptosis or aging. These responses can facilitate tumor suppression through preventing or repairing genomic damage, or by eliminating potential oncogenic cells in proliferating populations [61]. However, increasing evidence has demonstrated that p53 plays an important role in the metabolism of both normal and cancer cells. Abnormalities in its regulatory functions are closely related to various metabolic changes, particularly in tumor progression [62,63]. Recent research showed that p53 exhibited inhibitory effects on tumor proliferation by regulating the urea cycle [30]. 

In the liver, the UC is the major metabolic route converting excess nitrogen into urea. Recent studies have revealed that the expression of the UC enzymes is changed in cancer. The overexpression of CPS1 is related to a poor prognosis in colon cancer [64,65]. In hepatocellular carcinoma, reduced expression of CPS1 is linked to hypermethylation of the promoter and is related to reduced survival and lymphatic invasion [66]. A recent study reported decreased OTC expression in HCC patients’ samples; OTC is one of the characteristic arginine metabolism genes in pediatric sarcomas, brain tumors, and acute lymphoblastic leukemia [67,68]. 

The overexpression of ARG1 is related to poor prognosis in patients with colorectal cancer [35]. The majority of cancers show changes in the expression of at least two urea cycle enzymes that redirect nitrogen toward anabolic pathways, thereby sustaining tumor proliferation and invasion [69]. Therefore, controlling multiple components of the urea cycle may be helpful for p53′s powerful effects on ammonia metabolism that permit tumor inhibition, emphasizing the key role of the UC and ammonia clearance in tumorigenesis and their latent potential as therapeutic markers. 

Our results show that metformin inhibited the proliferation of HCT116 and SW480. After metformin treatment, the levels of urea cycle intermediate products decreased, and putrescine production was reduced both in vivo and in vitro, indicating that the UC and putrescine production may be inhibited. We observed a surprising inconsistency in the changes in glutamate: in the metformin group, increased glutamate was detected in vitro, while the level decreased in vivo. In rapidly proliferating cells, glutamine is used as a carbon source to supply the TCA cycle to sustain cellular bioenergetics and anabolic reactions. Previous research results indicate that, in cancer patients, certain tumors consume large amounts of glutamine, thereby reducing glutamine levels [34,70]. This may be why increased glutamate was observed in HCT116 cells that were administered metformin. We do not know the precise mechanisms underlying the differing production of glutamate in vivo and in vitro; we speculate that the decrease in the glutamate levels in the tumor tissues of mice in the metformin-treated group may have been caused by the duration of the drug treatment and the difference in the internal and external environments. Downregulation of the expression of the UC enzymes CPS1, OTC, and ARG1 was detected in CRC cells and HCT116 xenografts. These results demonstrate that metformin could inhibit the UC of CRC in vivo and in vitro. The inhibition of UC may lead to a decrease in cytoplasmic ornithine, a specific substrate of ODC for producing putrescine, which results in the inhibition of cancer cell proliferation. Reduced expression of the ODC protein and decreased putrescine were detected in both HCT116 xenografts and HCT116 cells after metformin treatment. These results prove that metformin inhibited colorectal cancer cell proliferation, and the mechanism may be related to downregulating the urea cycle enzymes and reducing polyamine biosynthesis. 

Inhibiting the urea cycle increases ammonia levels [30]. Li et al. found that ammonia influenced polyamine biosynthesis by restraining ODC activity [30]. Ammonia is a universal product of cell metabolism. In the microenvironments of tumors, accumulated ammonia is utilized for amino acid composites by cancer cells in vivo. Those biosynthetic pathways are sustained in both the tumor’s autonomous metabolism and systemic metabolism. The metabolic recycling of ammonia provides a key nitrogen source for breast cancer biomasses [71]. In this study, the expression of UC enzymes in HCT116 and SW480 cells was reduced after metformin treatment, and the inhibition of the UC resulted in the accumulation of ammonia. The 5 mM NH_4_Cl treatment restrained the proliferation of HCT116 and SW480 cells and decreased intracellular ODC expression. This may be because metformin inhibits the UC in CRC cells via AMPK–p53 signaling, leading to increased ammonia levels; in return, the increased ammonia further inhibits the UC. In conclusion, we speculate that the inhibition of colorectal cancer cell proliferation by metformin may be related to the inhibition of the urea cycle and decrease in ODC protein expression.

Our research revealed the relationship between metformin and the urea cycle change in colorectal cancer, which is expected to provide a new mechanism for tumor treatment. Unfortunately, few observations have been made on the changes in the UC in clinical samples from colorectal cancer patients. We will continue to study the urea cycle proteins and putrescine-producing proteins in clinical samples of colorectal cancer.

## 4. Materials and Methods

### 4.1. Reagents and Antibodies

Metformin Hydrochloride (purity: 99.98%, Cat.no.: HY17471A) and Nutlin-3 (purity: 98.32%, Cat.no.: HY-50696) were purchased from MedChemExpress (Monmouth Junction, NJ, USA). NH_4_Cl (purity: ≥99.5%, Cat.no.: A9434) and Pifithrin-α (purity: ≥95%, Cat.no.: P4359) were obtained from Sigma-Aldrich (St Louis, MO, USA). Chloroquine diphosphate (purity: ≥99%, Cat.no.: 4109) was purchased from Tocris Bioscience (Minneapolis, MN, USA). The antibodies against liver Arginase (ab91279), Ornithine Decarboxylase/ODC (ab97395), and Ornithine Carbamoyl-transferase/OTC [EPR19725] (ab203859) were obtained from Abcam (Cambridge, CB20AX, Cambridge, UK). 

Antibodies against p53 (**#** 2524S), AMPKα (#2532S), and β-actin (8H10D10) were obtained from Cell Signaling Technology (Danvers, MA, USA). Antibodies against phospho-AMPK alpha (Thr172) (AF3423) and phospho-p53 (Ser15) (AF3075) were purchased from Affinity Biosciences (Cincinnati, OH, USA). The Cell Counting Kit-8 was purchased from Dojindo (Mashikimachi, Kumamoto, Japan). Antibodies against p21(AP021) and LC3B(AL221) and the BeyoClick™ EdU Cell Proliferation Kit with Alexa Fluor 488 were purchased from Beyotime Institute of Biotechnology (Shanghai, China).

### 4.2. Cell Culture and Culture Conditions

HCT116 cells were purchased from the Shanghai Zhong Qiao Xin Zhou Biotechnology Company (Shanghai, China). SW480 cells were stored in the human tissue embryonic stem cell laboratory of Chongqing Medical University. The cells were incubated with Dulbecco’s Modified Eagle’s Medium (DMEM, Gibco-Invitrogen Corporation, Carlsbad, CA, USA), which contained 10% fetal bovine serum (FBS, Gibco-Invitrogen Corporation) and 1% antibiotic (Beyotime Institute of Biotechnology) in a humidified atmosphere with 5% CO_2_ at 37 °C.

### 4.3. Tumor Xenograft Models in Nude Mice

All the experimental procedures and protocols involving mice were authorized by the Experimental Animal Center of Chongqing Medical University. Five-week-old BALB/c nude mice were obtained from the Experimental Animal Center of Chongqing Medical University. Briefly, HCT116 cells (3 million cells suspended in 0.2 mL of PBS) were injected into the right armpits of nude mice as previously described [72]. 

Tumors were observed 8–10 days later, and 200 mg/kg/day of metformin dissolved in 200 μL of normal saline (NS) or 200 μL of NS was administered to the experimental or control group by gavage. The tumors were measured with a caliper and weighed every day. After four weeks, the mice were executed by dislocation after narcosis (Sigma, St. Louis, MO, USA), and the blood and tumors of all the mice were gathered for the later experiments. Each experiment was repeated three times.

### 4.4. Cell Viability Assay

The cell viability was determined using a Cell Counting Kit-8 (CCK-8) [73]. Briefly, according to the producer’s directions, cells at a density of 7 × 10^3^ per well were placed in 96-well plates for 24 h. The medium was replaced with fresh agent with metformin with distinct contents, and the cells were cultured for another 24 or 48 h. Later, the cell viability was determined using the CCK-8 assay. Before examining, CCK-8 solution (10 µL) was added to every well with a 100 µL mixture of culture medium. Then, the plates were cultured for 2 h in the incubator at 37 °C. Cell viability was calculated based on the absorbance at 450 nm by using an automatic microplate reader (Bio-Rad, Hercules, CA, USA). The OD_450_ value corresponded to the cell viability [74]. Each experiment was repeated three times.

### 4.5. Cell cycle Analysis

HCT116 and SW480 cells were plated in six-well plates at a density of 3 × 10^5^ mL^−1^. After 24 h, the cells were exposed to metformin at different concentrations (0 or 20 mM) or Nutlin-3 at a concentration of 0 or 5 µM and NH_4_Cl (0 or 5 mM) for another 24 h. We gathered 2 × 10^6^ cells in triplicate. After cleaning twice using cold PBS, we resuspended the cells in 75% ethanol and fixed them overnight at 4 °C. Next, the fixed cells were cleaned with PBS and cultured with RNase A and PI in the absence of light for 30 min at 37 °C. Then, the cell cycle was analyzed with FAC-Scan laser flow cytometry. The data were analyzed using the CELL Quest software (1.1 version, BD Biosciences, Franklin Lakes, NJ, USA) [73]. Each experiment was repeated three times.

### 4.6. Colony-Formation Assay

The colony-formation assay was performed as previously detailed [73]. Briefly, HCT116 and SW480 cells were seeded in plates 60 mm in diameter at a density of 1 × 10^3^ and treated with or without metformin for 24 h. The cells were cultured for 16 days in metformin-free conditions. The colonies were fixed with 4% paraformaldehyde for 30 min at room temperature. The cells were cleaned with PBS twice and then stained with Wright–Giemsa solution (Beyotime Institute of Biotechnology, Shanghai, China). The plates were imaged, and the number of colonies was counted. Each experiment was repeated three times.

### 4.7. Immunoblot Assay

The immunoblot assay was performed as previously detailed [74]. Briefly, the cells were separated and cleaned with cold PBS and suspended again in lysis solution with 1% phenyl-methane-sulfonyl fluoride and phosphatase inhibitor supplemented immediately before use (Beyotime Institute of Biotechnology, Shanghai, China). After resting on ice for 30 min, the supernatant was gathered after centrifugation for 15 min at 4 °C at 12,000 rpm, and the protein content was evaluated using a BCA Protein Assay Kit (Beyotime Institute of Biotechnology). 

Equal amounts of proteins from every sample were segregated through sodium dodecyl sulfate–polyacrylamide gel electrophoresis (SDS-PAGE), and the segregated proteins were transferred to polyvinylidene difluoride (PVDF) membranes. The membranes were incubated with 5% fat-free milk for 2 h and then exposed to primary antibody at 4 °C overnight, before being cleaned in Tris-buffered saline with Tween20 (TBST) for 30 min, and incubated with IgG horseradish peroxidase (HRP)-conjugated secondary antibody for 1 h at room temperature. The bound immune complexes were visualized using enhanced chemiluminescence (ECL) Western blotting detection reagents (Millipore, Billerica, MA, USA) and the Luminescent Image Analyzer (Bio-Rad). Each experiment was repeated three times.

### 4.8. EdU Proliferation Assay

The EdU proliferation assay was performed as previously detailed [73]. Briefly, cells were plated in 24-well plates and cultured under the specified conditions overnight. After 24 h, we treated the HCT116 and SW480 cells with or without metformin (20 mM), or with or without Nutlin-3 (5 µM) for 24 h.

The cell proliferation was assessed using the incorporation of EdU with the BeyoClick™ EdU Cell Proliferation Kit with Alexa Fluor 488. The cells were exposed to 10 µM EdU for 2 h before fixation, permeabilization, and EdU staining, which were performed in line with the manufacturer’s instructions. The cell nuclei were colored using Hoechst 33,342 for 20 min. The ratio of cells that had incorporated EdU was determined with a fluorescent microscope. Each experiment was repeated three times.

### 4.9. LC-MS Analysis for Metabolites

For the tissue samples, 60 mg of HCT116 xenografts were sampled, and each sample was precooled using 200 µL of ultra-pure water for MP homogenization treatment. For the cell samples, HCT116 cells were seeded in 6-cm dishes and cultured overnight. After 24 h, we treated the cells with metformin (0 or 20 mM) for 24 h. Then, the cells were harvested for metabolite extraction. We took each sample, added 500 μL of precooled 0.3% ammonium acetate solution, washed the sample twice, and then quenched with liquid nitrogen. 

We added precooled MeOH/ACN/H2O (2:2:1, *v*/*v*/*v*) to the samples, and then scraped off the cells and transferred them to a 1.5 mL centrifuge tube. We then vortexed the samples for 30 s and sonicated them in ice for 20 min. To precipitate the proteins, the samples were incubated for 1 h at 20 °C, followed by 20 min of centrifugation at 14,000 rpm and 4 °C. Then, the resulting supernatant was taken into a normalized volume and evaporated to dryness in a vacuum concentrator. 

The dry extracts were then reconstituted in 100 mL of ACN: H_2_O (1:1, *v*/*v*), sonicated for 10 min, and centrifuged for 15 min at 13,000 rpm and 4 °C to remove insoluble debris [75]. The amino-enone derivatives were identified and quantified by liquid chromatography coupled to tandem mass spectrometry (LC-MS/MS) [76]. The Agilent 1290 Infinity LC equipped with a Zic-HILIC column (3.5 µm, 2.1 mm ×150 mm) was employed for the high-resolution separation of amino acids. The injection volume was 2 µL. 

The mobile phase consisted of eluent A (25 mM HCOONH_4_ with 0.08% HCOOH) and eluent B (acetonitrile with 0.1% HCOOH), and separation was achieved using a linear gradient of eluent B (0–12 min, 90% B to 70% B; 12–18 min, 70% B to 50% B; 18–25 min, 50% B to 40% B; and 25–30 min, 40% B) at a flow rate of 250 µL/min. The column was maintained at 40 °C. All the mass spectra were acquired using a mass selective detector (5500 QTRAP) coupled with an electrospray ionization (ESI) source, with a condition of a 500 °C source temperature, 40 ion Source Gas1 (Gas 1), 40 Ion Source Gas2 (Gas 2), 30 Curtain gas (CUR), and 5500V ion Sapary Voltage Floating (ISVF). 

Mass spectra from positive-ion ESI were recorded over the range of 50–2200 *m*/*z*. The MRM mode was used to detect the ion pairs of all the amino-enone derivatives. The area of the base-peak and the retention time were extracted using the Multiquant software to identify the metabolite. The standard for amino-enone derivatives was used to correct the retention time, while the retention time and the area of the base-peak were extracted using the Multiquant software to identify the metabolite. Boxplots of amino acids were generated using an R package. 

### 4.10. Statistical Analysis

The figures in the text were compiled from at least three separate experiments. The data are expressed as the means ± SDs (standard deviations). All the statistical analysis was performed and P values were calculated using the GraphPad Prism software 7.0 (GraphPad Software, Inc., San Diego, CA, USA) and are indicated as * *p* < 0.05, ** *p* < 0.01, and *** *p* < 0.001.

## 5. Conclusions

In summary, our results demonstrate that metformin could remarkably inhibit the proliferation of colorectal cancer cells, which may be associated with the inhibition of the urea cycle and reduction of putrescine generation in vitro and in vivo.

## Data Availability

The data presented in this study are included in this published article.

## Sample Availability

Samples of the compounds are not available from the authors.
